# Citation success of different publication types: a case study on all references in psychology publications from the German-speaking countries (D–A–CH–L–L) in 2009, 2010, and 2011

**DOI:** 10.1007/s11192-015-1573-y

**Published:** 2015-04-01

**Authors:** Günter Krampen, Peter Weiland, Jürgen Wiesenhütter

**Affiliations:** 1 Leibniz Institute for Psychology Information (ZPID), Trier, Germany; 2 Department of Psychology, University of Trier, Trier, Germany

**Keywords:** Citation analysis, Publication success, Publication type, Publication genre, Bibliometry, Scientometry, Psychology

## Abstract

Scientometric data on the citation success of different publication types and publication genres in psychology publications are presented. Data refer to references that are cited in these scientific publications and that are documented in PSYNDEX, the exhaustive database of psychology publications from the German-speaking countries either published in German or in English language. Firstly, data analyses refer to the references that are cited in publications of 2009 versus 2010 versus 2011. With reference to all cited references, the portion of journal articles ranges from 57 to 61 %, of books from 22 to 24 %, and of book chapters from 14 to 15 %, with a rather high stability across the three publication years analysed. Secondly, data analyses refer to the numbers of cited references from the German-speaking countries, which are also documented in PSYNDEX. These compose about 11 % of all cited references indicating that nearly 90 % of the references cited are of international and/or interdisciplinary publications not stemming from the German-speaking countries. The subsample shows the proportion of journal articles, books, and chapters, and these are very similar to the percentages identified for all references that are cited. Thirdly, analyses refer to document type, scientific genre, and psychological sub-discipline of the most frequently cited references in the psychology publications. The frequency of top-cited references of books and book chapters is almost equal to that of journal articles; two-thirds of the top-cited references are non-empirical publications, only one-third are empirical publications. Top-cited references stem particularly from clinical psychology, experimental psychology, as well as tests, testing and psychometrics. In summary, the results point to the fact that citation analyses, which are limited to journal papers, tend to neglect very high portions of references that are cited in scientific publications.

## Introduction

Limitations of the databases that are used in citation analyses hinder and complicate evaluations of the citation success of different scientific publication types (e.g., books, chapters, journal articles, test manuals, and therapy manuals) and publication genres (e.g., theoretical, methodological, or empirical studies, literature reviews/overview, textbooks, monographs, etc.). This can result in serious unfairness in scientometric evaluations of individual scientists, research teams, departments, institutions, and nations as well, because some of their scientific and scholarly work is underrepresented or—even worse—neglected.

This problem applies—for example—to textbooks and teaching materials published in book chapters, because these publications appertain to the scholarly performance of scientists, which report and integrate primary research results and theory building in (more or less) creative and—more important—didactically clear, structured form, which is motivating for students in education and further training. At least in medicine and psychology, this applies also to test manuals, therapy and prevention manuals as well as diagnostic and therapeutic guidelines, because of their outstanding significance not only for education and training, but also for applied diagnostic and therapeutic work in medical and psychotherapeutic treatments. Thus, these scientific publication types and publication genres must be considered in evaluations of scientists and science as well as primary research reports. Besides other criteria and indicator variables of scientific productivity and performance (e.g., research funding, invited talks at international congresses, innovations, and letter patents), this belongs to the criteria of scientific and scholarly publication output which constitute multiple bibliometric indicators and—if cited—scientometric indicators of productivity and its impact in the sciences (see, e.g., Glänzel and Moed 2013; Moed and Halevi 2014).

The problem is that the selectivity of international and interdisciplinary databases (e.g., *Web of Science* and *Scopus*) that are used in citation analyses hinders evaluations of the citation success of different scientific publication types (e.g., books, chapters, journal articles, test manuals, and therapy manuals) and publication genres (e.g., theoretical, methodological, or empirical studies, literature reviews/overview, textbooks, monographs, etc.), and this is a situation which can result in serious unfairness in scientometric evaluations. For example, the *Web of Science* (*WoS*) and *Scopus*, surely the most frequently used citation databases, pushes journal articles (mainly English-language papers from the Anglo-American research community; see, e.g., Albarrán et al. 2010; González-Alcaide et al. 2012; Krampen 2009) and ignore widely—with very few and really only English-language exceptions—books, book chapters, test manuals, and intervention (therapy) manuals in its documentation of publications and of reference lists (see, e.g., Chi 2014; Ossenblok et al. 2013; van Leeuwen 2013). More than that, comparative scientometric results document significant differences in the coverage, e.g., of *WoS* and *Scopus* (Alves et al. 2014) and others as well (see, e.g., Cavacini 2015, for computer sciences).

At the other extreme, *Google Scholar* checks the “complete” Internet for citations of publication titles (or author names) including all and perhaps nothing, because the references identified refer not only to citations in scientific publications, but to these at homepages of individuals, institutions, and organizations, in excerpts, homework of high school and college students, examination schedules and exam papers, self-presentations, advertising and advertisements, etc. without any type of quality control. Therefore, citation success according to *Google Scholar* can be manipulated very easily and, therefore, can be considered poor and not very useful for scientometrics (see, e.g., Aguillo 2012; Beel and Gipp 2010; Mayr and Walter 2007; Mingers and Lipitakis 2010; for a recent discussion of the weaknesses of *Google Scholar* and its comparison with the *WoS* see, e.g., de Winter et al. 2014). In addition, there are huge problems with homonyms and identical or similar titles of publications. Somewhat in between of the high selective, restrictive *WoS* and the not selective, but complete open *Google Scholar* without any professional specialist quality control and scientific quality management are databases like *Ovid*, which are dominated by publishers and/or database providers because they tend to selectively include primarily their “own” products (see, e.g., Larsen and von Ins 2010; Mingers and Lipitakis 2010). Thus, the argument is that these are selective, or bad quality or publisher controlled databases but yet no complete, open, quality databases.

Therefore, for more profound and serious citation analyses we need databases that cover all types and genres of scientific and scholarly publications including their complete reference lists. Because of a better manageable population of scientists and their publications it is more feasible to work first of all with local and disciplinary databases, which may be merged or combined to international multidimensional databases subsequently. Citation analyses, which refer to such exhaustive databases, can show what proportions of publication output are neglected in citation analyses, which refer to the multidimensional, international databases (as the *WoS, Scopus,* etc.; see, e.g., Hicks 2005, on publication types; Chi 2014, on publication languages). Furthermore, it can be expected that the results of such citation analyses will have better acceptance not only by the evaluators but also and especially by the evaluated scientists, that is, by the scientific community.

Since the publication year 2009, PSYNDEX, the database of psychology publications from Austria, Germany, Liechtenstein, and the German-speaking regions of Luxemburg and Switzerland published either in German or in English language, includes the complete reference lists of publications documented. Thus, PSYNDEX is a local disciplinary database for psychology and its neighbouring disciplines. Access to PSYNDEX is provided, e.g., by the platforms Ovid and Ebsco as well as www.zpid.de, open access is possible for individual users in PubPsych (http://www.zpid.de/index.php?wahl=products&uwahl=pubpsych).

PSYNDEX is a domain-specific literature database which covers the scientific literature of a discipline better than citation databases such as the multidisciplinary *WoS* (coverage ≤20 % of the PSYNDEX-documents in the WoS; see, e.g., Krampen 2009), for which Larsen and von Ins (2010) report in addition a general decline in coverage of scientific publications. A special feature of PSYNDEX is its completeness for the psychology publication output of its defined area, that is, the German-speaking countries of the world, which have sometimes been called the “D–A–CH–L–L” countries (D = Germany, A = Austria, CH = Switzerland, L = Luxembourg, L = Liechtenstein). Thus, the following scientometric analyses refer to all the references that are cited in the psychology publications documented in PSYNDEX for the publication years 2009, 2010, and 2011. Analyses are conducted separately for the references cited in the publications documented in PSYNDEX with the publication year (1) 2009, (2) 2010, and (3) 2011. This has the advantage that cross-sectional time comparisons are possible thus allowing conclusions to be made on the stability of the results.

Main objective of our scientometric study is to analyse the frequencies by which different publication types and publication genres are cited. Because this question can not be answered up to now by the use of multidisciplinary international databases (focus at journals, neglect or even ignorance of book chapters, books, test manuals, etc.), we conduct a case study with the local disciplinary databases PSYNDEX. The *first research question* refers to (1) the frequencies of different publication types (i.e., journal articles, books, book chapters) to which reference is given by citations in the publications of the years 2009, 2010, and 2011. The *second research question* refers to (2) the replication of the first analysis; however, data refer only to the cited references in publications from the German-speaking countries that are also documented in PSYNDEX (11 % of all cited references) and whose PSYNDEX documentation contains meta-data about the publication genre and on the psychological sub-discipline to which they belong. The *third research question* refers to (3) the characteristics of the most frequently cited references in the psychology publications from the German speaking countries published in 2009, 2010, and 2011. These top-cited references are analysed with the help of PSYNDEX meta-data for the relative frequencies of (3a) different document types, i.e., journal articles, books, chapters, (3b) different scientific publication genres, i.e., empirical and non-empirical publications, and (3c) different psychological sub-disciplines following the classification codes (CC) of the *Thesaurus of Psychological Index Terms* of the *American Psychological Association* (APA; Gallagher Tuleya 2007), i.e., methodology, basic psychology, and applied psychology, etc. In addition, the citation success over time of the top-cited publications (see, e.g., Johnston et al. 2013) is briefly described.

## Methods

For the scientometric analysis, we use the raw frequencies of the documents included in PSYNDEX published in 2009, 2010, and 2011. PSYNDEX includes 7358 documents with reference lists published in 2009, 8484 documents published in 2010, and 8059 documents published in 2011. For the citation analysis, we use the reference lists of these documents, which sum up to 402,830 references cited in 2009, 426,014 references cited in 2010, and 475,935 references cited in 2011. PSYNDEX and PubPsych documents include various meta-data (e.g., full bibliographic specification, publication type, controlled method, abstract, descriptors, classification categories, etc.; Gallagher Tuleya 2007; ZPID 2011) and the complete reference lists of publications documented.

## Results

### Number and publication types of all the references cited

Mean number of references cited per publication is *M* = 54.7 in 2009, *M* = 50.2 in 2010, and *M* = 59.0 in 2011; all average values with very large ranges reaching from zero to several thousands references cited in one publication. The very long references lists are typical for textbooks and handbooks as well as—at least partially—for dissertation theses and some monographs.

The distributions of the references cited in the 2009, the 2010, and the 2011 publications across different document/publication types (DT) are reported in the upper part of Table 1. Absolute and relative frequencies show that approximately 60 % of the references cited are journal articles, around 23 % are books, and 15 % are book chapters. The rest of the “other” references cited could not be automatically identified because information such as title of publication, journal title, editors, and/or page numbers is missing. Results point to a rather time-stable pattern of this distribution across different publication types and to the fact that citation analyses, which concentrate on journal articles, neglect citations of approximately 40 % of the scientific and scholarly publication output in psychology, i.e., all citations of books, book chapters, test manuals, etc. This result shows that recent psychology is definitely neither a “journal science” (alike the natural sciences) nor a “book science” (alike the humanities and social sciences; see, e.g., Wray and Bornmann 2015), but just in between of these two scientific publication cultures.

**Table 1:** Document type (DT) of cited references in the psychology publications from the German-speaking countries published in 2009, 2010, and 2011

Document type (DT) of the cited references	Number (N) and publication year (PY) of the citing publications					
	*N* = 7758 in 2009		*N* = 8484 in 2010		*N* = 8059 in 2011	
	*f*	%	*f*	%	*f*	%
All cited references	402,830		426,014		475,935	
Journal articles	230,923	57.3	258,724	60.7	290,783	61.1
Books	97,402	24.2	94,377	22.2	103,644	21.8
Book chapters	62,225	15.4	60,634	14.2	67,397	14.2
Other^a^	12,280	3.1	12,279	2.9	14,111	2.9
Number of cited references from the German-speaking countries, that are documented in PSYNDEX	46,819	11.6^b^	46,179	10.8^b^	53,627	11.3^b^
Journal articles	27,061	57.8	28,008	60.7	33,246	62.0
Books	10,020	21.4	9007	19.5	9861	18.4
Book chapters	8488	18.1	7980	17.3	9171	17.1
Other^c^	1250	2.7	1184	2.5	1349	2.5

^a^DT not automatically identifiable by search routine

^b^Percentage (%) of all cited references (see above, first line of Table)

^c^Institutional reports, audio-visual media, addenda, dissertations

### Number and publication types of the references cited and documented in PSYNDEX

The subsets of references cited from the German-speaking countries, which are also documented in PSYNDEX, are presented in the lower part of Table 1. These compose approximately 11 % of all cited references (for the percentages per publication year see Table 1). This result reveals that nearly 90 % of the references cited in publications documented in PSYNDEX are international and/or interdisciplinary publications not originating from the German-speaking countries (because they are not documented in PSYNDEX).

Specifically, the proportions of journal articles (around 60 %), books (around 20 %), and book chapters (around 17 %) in these subsets are very similar to the percentages identified for all references that are cited (see above and contrast the upper and the lower parts of Table 1). We can conclude that these subsets of cited references are representative for all references cited with reference to their distribution across different publication types. Therefore, we can use the subsets for more detailed scientometric analyses with the help of meta-data available in the PSYNDEX documents. We can even use the percentages of the cited references categorized as “other” for further scientometric analysis, because they can be identified with the help of PSYNDEX meta-data as citations of institutional reports, audio-visual media, addenda, and dissertations.

### Features of the top-cited references in 2009, 2010, and 2011 publications

We use the frequency *f* > 14 citations per publication year as definition of top-cited references because the commonly used top-100 references definition led to an inflation of bounded rank positions. The *f* > 14 criteria led to the top-65 cited references in the publication year PY = 2009, the top-61 cited references in PY = 2010, and the top-93 cited references in PY = 2011. The rise of the number can not be explained by the growth of the publication sets in the PY, because it is random and—therefore—can not be the explanation for the slight differences in the cited time window reported in the next paragraph. Since also the popular top-100-criteria is somewhat arbitrary and proved (because of an inflation of bounded rank positions) not to be very selective, we think the *f* > 14 criterion is justifiable in an exploratory approximation.

#### Time window of top-cited references in 2009, 2010, and 2011 publications

**Fig. 1:**
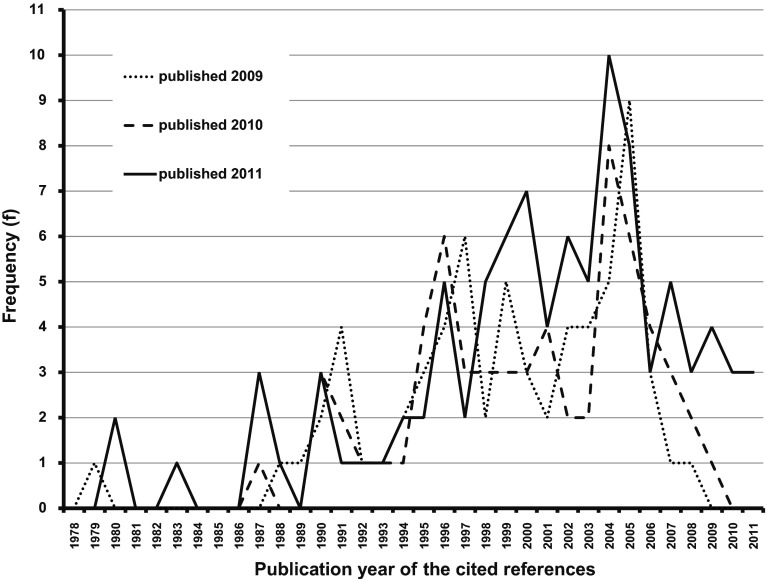
Publication years of the most frequency cited references (*f* > 14) in psychology publication from the German-speaking countries in 2009, 2010, and 2011

The frequency distributions across the publication years of the most frequently cited references in psychology publications from the German-speaking countries in 2009, 2010, and 2011 are graphically presented in Fig. 1. All three frequency distributions are skewed to the left, which points to the result that recent publications dominate the reference lists. Oldest references cited go back to the late 1970s to the late 1980s. In comparison with the humanities this indicates a rather weak historical citation pattern and in comparison to the natural sciences, however, a quite long time window—at least within the top-cited references in psychology.

Most top-cited references in the 2009 publications stem from a rather narrow time frame of only 4 years (2001–2005; see Fig. 1). The time frame becomes somewhat broader for the top cited references in the 2010 publications (6 years from 2001 to 2006) and in the 2011 publications (9 years from 1999 to 2007), but remains rather limited to recent publications of <10 years before the publication. With the exception of a slight trend to more recently published references ranging from about 2 years prior to or even in the year of the publication of the citing paper in the publication year 2011, these results are rather stable over time. Overall, the results are in good agreement with the descriptions of a narrow time frame (6–7 years) for the references cited that was verified by Johnston et al. (2013) for other sciences than psychology.

#### Publication type and publication genre of top-cited references in 2009, 2010, and 2011 publications

The distributions across different publication types and across different publication genres of the most frequently cited references in psychology publications from the German-speaking countries in 2009, 2010, and 2011 are presented in Table 2. Within the top-cited references in all three publication years analysed here, the frequencies of the *publication/document types* (*DT*) of books and book chapters on the one side and journal articles on the other side are represented very similarly (see Table 2, upper part). On average, the proportion is 50:50 with only a slight trend in favour of journal articles in the 2010 and 2011 publications. With reference to the recent debate in the psychology research community in the German-speaking and other non-English-speaking countries (for a summary, see, e.g., Bornmann et al. 2012 or Krampen 2009; see in addition, e.g., González-Alcaide et al. 2012), it is of interest that the number of English-language journal articles that are the top cited markedly exceeds the number of German-language journal articles that are top cited (from all the German-speaking countries).

**Table 2:** Document type (DT) and scientific genre (controlled method, CM) of the most frequently cited references (*f* > 14 citations) in the psychology publications from the German-speaking countries published in 2009, 2010, and 2011

Characteristics of the most frequently cited references	Most frequently cited references (*f* > 14) in psychology publications from the German-speaking countries in the publication years (PY)					
	PY = 2009^a^		PY = 2010^b^		PY = 2011^c^	
	*f*	%	*f*	%	*f*	%
Document type (DT)						
Books (all German-language)	26	40	21	34	30	32
Editions (all German-language)	4	6	1	2	0	0
English-language chapters	2	3	4	7	5	5
German-language chapters	2	3	2	3	3	3
Σ books and book chapters		52		46		41
English-language journal articles	22	34	26	43	42	45
German-language journal articles	9	14	7	11	13	14
Σ journal articles		48		54		59
Scientific genre/controlled method (CM)						
Textbooks/handbooks	14	22	9	15	15	16
Test manuals	2	3	2	3	4	4
Intervention (therapy) manuals	5	8	3	5	3	3
Literature overviews/reviews	17	26	17	28	28	30
Theoretical studies	6	9	7	11	10	11
Σ non-empirical publications		68		62		65
Methodological studies	3	5	4	7	6	6
Empirical (field) studies	10	15	12	20	15	16
Experimental studies	4	6	4	7	10	11
Meta-analyses	4	6	3	5	2	2
Σ empirical publications		32		38		35

^a^ *N* = 65 most frequently cited references (*f* > 14 citations) in publications of 2009

^b^ *N* = 61 most frequently cited references (*f* > 14 citations) in publications of 2010

^c^ *N* = 93 most frequently cited references (*f* > 14 citations) in publications of 2011

The distribution of the top-cited references across *different publication genres* (i.e., controlled method, CM, according to the APA Thesaurus of Psychological Index Terms; Gallagher Tuleya 2007) is presented in the lower part of Table 2. A very consistent finding across the three publication years under study is that the non-empirical publications dominate in the top-cited references with an average proportion of 66 %, that is, two-thirds. Most represented genres are literature reviews, overview as well as handbooks and textbooks, and somewhat less in focus are theoretical studies and manuals. Empirical publications compose one-third of the top-cited references with empirical (field) studies (i.e., correlation studies, questionnaire studies, pre-experimental studies, etc.) at the top of this list. A striking result is that experimental studies are equally seldom found in the top-cited references as methodological studies and meta-analyses (see Table 2).

#### Psychological sub-disciplines of top-cited references in 2009, 2010, and 2011 publications

The distributions across the different psychological sub-disciplines (*Classification Codes,* CC, in the APA-*Thesaurus of Psychological Index* Terms; Gallagher Tuleya 2007; ZPID 2011) of the most frequently cited references in the psychology publications from the German-speaking countries published in 2009, 2010, and 2011 are presented in Table 3. The results show that top-cited references particularly stem from and belong to the comparable larger research domains of clinical psychology and experimental psychology with more personnel and research funding resources as well as more publication media than other psychological sub-disciplines.

**Table 3:** Psychological subdiscipline (classification category, CC) of the most frequently cited references (*f* > 14 citations) in the psychology publications from the German-speaking countries published in 2009, 2010, and 2011

Classification category of the most frequently cited references (CC)^d^	Most frequently cited references (*f* > 14) in psychology publications from the German-speaking countries in the publication years (PY)					
	PY = 2009^a^		PY = 2010^b^		PY = 2011^c^	
	*f*	%	*f*	%	*f*	%
Tests and testing, psychometrics	10	15	8	13	12	13
Statistics and mathematics	2	3	4	7	5	5
Research methods and experimental design	4	6	2	3	4	4
Σ Methodology		25		23		22
Human experimental psychology	11	17	11	18	18	19
Physiological psychology and neuroscience	2	3	1	1	8	9
Developmental psychology	6	9	4	7	6	6
Social psychology	1	1	2	3	3	3
Personality psychology	1	2	2	3	2	2
Σ Basic psychology		32		32		39
Clinical psychology	22	34	20	33	25	27
Educational psychology	1	2	4	7	2	2
Industrial and organizational psychology	2	3	3	5	7	8
Σ (Larger) applied psychologies		39		45		37
Various CC						
History and systems	0		0		0	
Psychology and the humanities	0		0		0	
Communication systems	0		0		0	
Social processes and social issues	3	5	0	0	1	1
Animal (…) psychology	0		0		0	
(Specific) applied psychologies						
Sport psychology and leisure	0		0		0	
Military psychology	0		0		0	
Consumer psychology	0		0		0	
Engineering and environmental psychology	0		0		0	
Intelligent systems	0		0		0	
Forensic psychology and legal issues	0		0		0	

^a^ *N* = 65 most frequently cited references (*f* > 14 citations) in publications of 2009

^b^ *N* = 61 most frequently cited references (*f* > 14 citations) in publications of 2010

^c^ *N* = 93 most frequently cited references (*f* > 14 citations) in publications of 2011

^d^Classification categories (CC) according to the *APA thesaurus of psychological index terms*

At first glance this result seems to be rather trivial. However, it must be noted that the proportions of experimental psychology publications as well as publications on tests, testing, psychometrics, and test development in the top-cited references are higher than is expected with reference to the complete relative publication output of these sub-disciplines: For example, the expected proportion (*p*_e_) of all experimental psychology publications documented in PSYNDEX is *p*_e_ = 10.4 %, their observed proportion (*p*_*o*_) in the top-cited references is *mean p*_o_ = 18.0 % (see Table 3). With reference to the complete PSYNDEX database, the expectancy value for publications on tests and psychometrics is *p*_e_ = 5.5 %, their observed proportion in the top-cited is around *mean p*_o_ = 14.0 %. At a lower level this is similar for psychology publications on statistics and mathematics (*p*_e_ = 1.3 %; *mean p*_o_ = 5.0 %). Thus, in comparison to the publication output of experimental psychology, of research on tests and psychometrics as well as of psychology publications on statistics and mathematics, these sub-disciplines are visibly overrepresented in the top-cited references.

Contrary to these results for publications on experimental psychology, tests and psychometrics as well as on statistics and mathematics are the relations between the expected and in the top-cited observed relative frequencies for publications on clinical psychology (*p*_e_ = 46 %; *mean p*_o_ = 31 %), educational psychology (*p*_e_ = 9 %; *mean p*_o_ = 4 %), industrial and organizational psychology (*p*_e_ = 8 %; *mean p*_o_ = 5 %), personality psychology (*p*_e_ = 6 %; *mean p*_o_ = 2 %) as well as on social processes and social issues (*p*_e_ = 13 %; *mean p*_o_ = 2 %). Thus, in comparison to their publication output, publications on these psychological sub-disciplines are markedly underrepresented in the top-cited reference lists. For completion, it is reported that the relations between expected relative frequencies and the observed relative frequencies in the top-cited reference lists are balanced for publications on research methods and experimental design, physiological psychology and neuroscience, developmental psychology as well as social psychology (see Table 3). All other classification categories (CC) are not represented in the top-cited reference lists (see lower part of Table 3); however, their portions of the complete publication output documented in PSYNDEX are rather small.

Last, but not the least, it should be noted that—summed up—the applied psychological sub-disciplines possess the highest proportions at the top-cited references (*mean p*_o_ = 40 %); nevertheless, this is less than is expected according to their proportions of the complete publication output (*p*_e_ = 63 %). The basic psychological sub-disciplines are ranked second (*mean p*_o_ = 34 %) and publications on methodology are ranked third (*mean p*_o_ = 23 %) in the top-cited references. Thereby, it must be considered that psychology publications dealing with the applied sub-disciplines and at least some of the basic sub-disciplines (e.g., developmental psychology and social psychology) are of special relevance in the education and training of many other professionals (e.g., teacher education students and teachers, medical personnel, management and marketing personnel, etc.). Thus, the publication output is rather high (and especially higher than the output of psychology publications on methodology, which are of primary interest for psychologists), but these publications are less cited in psychology publications because they address other target groups.

## Discussion and conclusions

The results of the present case study confirm serious deficits in citation analyses, which refer exclusively to selective databases including only journal articles, only publications of certain publishers or publishing groups or—at the other extreme—everything that can be detected on the World Wide Web. Our data refer to all the references cited in the scientific and scholarly psychology publications that are documented in PSYNDEX, the local disciplinary database of psychology publications from Austria, Germany, Liechtenstein, and the German-speaking regions of Luxemburg and Switzerland, in either the German or English language. With reference to all of the more than 1,303,000 cited references, the proportion of cited journal articles ranges from 57 to 61 %, of cited books from 22 to 24 %, and of cited book chapters from 14 to 15 %, with a rather high stability across the three different publication years analysed. Thus, evaluations of scientists and sciences, which rely solely or primarily journal articles including citation databases (like the *Web of Science* or *Scopus*), neglect at least about 40 % of the scientific and scholarly publications and result in extremely biased scientometric results that are unfair for individual scientists, scientific teams, departments, institutions, and nations.

It is noteworthy to see that about 90 % of all references in the German- and English-language publications from the German-speaking countries stem *not* from the German-speaking countries, but rather from the Anglo-American (most of them) or other countries. Thus, psychology in the D–A–CH–L–L states is a very strongly international oriented working and publishing research community. This scientometric result conforms to these on an increasing international collaboration (Kliegl and Bates 2011) and on the strong increase of English-language journal-publications in psychology in the German-speaking countries (Krampen et al. 2005; Michels and Schmoch 2014).

More detailed scientometric analyses were possible for the subset of references that are cited *and* are documented in PSYNDEX with their meta-data as well. This subset constitutes about 11 % of all cited references. The proportions of citations of journal articles, books, and chapters in this subset are very similar to the percentages identified for all references that are cited. Thus, this subset samples the complete references that are cited in a representative form.

A frequency of *f* > 14 citations per publication year turned out to be the best indicator for the most frequently cited top references in the three consecutive publication years under study. This criteria leads to top-65 cited references in the publication year PY = 2009, top-61 cited references in PY = 2010, and top-93 cited references in PY = 2011. Descriptive analyses of the frequency distributions across the publication years of these top-cited references resulted in graphs that are skewed to the right. This skewness reveals that recent publications dominate the reference lists. This result confirms the scientometric results of Bornmann et al. (2014) that the years shortly after publication are most significant for the later total citation impact of a publication.

The oldest cited references go back to the late 1970s, indicating in comparison with the humanities and social sciences a rather weak historical citation pattern—at least within the top-cited references. Most of the top-cited references in the publications stem from a rather narrow time frame of only 4–9 preceding years, with a slight trend toward citing more references published in the prior 2 years or even in the publication year of the citing paper in the publication year 2011. The result shows that in psychology the first years after publication are the decisive ones whether an article will be highly cited or not. Johnston et al. (2013) found similar patterns for top economic journals (6–7 years), hence psychology and economy seem to be similar in this regard.

Citation analyses with reference to a more detailed document type, to the scientific genre, and to the psychological sub-disciplines of the most frequently cited references in the psychology publications from the German-speaking countries published in 2009, 2010, and 2011 were possible with the help of the meta-data available in PSYNDEX. Regarding frequency, books and book chapters are represented almost as equally in the top-cited references as journal articles. Two-thirds of the top-cited references are not empirical publications (most frequently these are literature overviews/reviews and handbooks/textbooks); only one-third are empirical publications (mainly empirical field studies). This result on the publication success of different publication genres in psychology differs from that presented by Johnston et al. (2013) for the publication success in economics. The results of Johnston et al. (2013, p. 1023) indicate, “that empirical papers attract more citation success than theoretical studies”, a finding that is actually contrary to our scientometric results for psychology.

An a posteriori hypothesis for our results on the lower citation success of empirical publications in psychology may be the interpretation that empirical studies (in psychology) try to contribute to the solving of empirical problems or—at best—solve such a problem but nevertheless frequently conclude that “further research is needed”. This original empirical study will be cited in some (but rather few) subsequent empirical studies on the same research question, which—at best—advance the research and replace the earlier study, thus remaining only one link in a longer chain of empirical studies (perhaps with some historiographical significance), and in the reference lists of the following publications (maybe with the exceptions of some classics in empirical research). On the contrary, non-empirical publications (i.e., especially literature reviews, overviews, handbooks, and theoretical studies) describe, structure, integrate, and evaluate research in a broader problem domain where, at best, such publications identify significant topics for research, describe research deficits, and motivate (more or less) innovative research in a wider problem domain. This actually results in higher citation success, because many later publications can have direct access to the conclusions made in these non-empirical publications.

The results show that the top-cited references stem particularly from clinical psychology, experimental psychology, as well as from research on tests, testing and psychometrics. This covaries only partially with the size of the sub-disciplines’ scientific community in terms of personnel and research funding resources as well as publication media. Contrasts of the expected relative frequencies, which are computed with reference to the complete publication output of a sub-discipline and its proportion of indexed documents recorded in PSYNDEX with the observed relative frequencies in the top-cited reference lists, show that publications on clinical psychology, educational psychology, personality psychology, and research on social processes and social issues appear less frequently in the top-cited references than would be expected. This is contrary for the relative frequency of references given to publications on experimental psychology, on research on tests and psychometrics as well as on psychology publications on statistics and mathematics.

In summary, the results points to the fact that citation analyses, which are limited to journal papers, neglect very high proportions of references that are cited in scientific and scholarly publications. The same is valid for databases that concentrate on books and chapters—at least in psychology publications from the German-speaking countries. Professional—and perhaps special databases for scientific disciplines—databases with high quality control and high coverage of the publications and their complete reference lists are required for serious and reliable citation analyses, which result in fair evaluations of the citation success of scientists, research teams, institutions and nations. Such scientometric results will be accepted with a higher probability in the scientific community.

The focus on journals when using *WoS* or *Scopus* as databases for citation analysis in psychology not only misses a large part of publication output but the most important one: While books and book chapters account for 30 % percentage of the publication output in psychology, 52 % of the highly cited documents are books or book chapters. Hence, books and book chapters are still an important document type that must not be neglected. If evaluation focuses only on journal articles, most of the highly cited, i.e., important, works are not taken into account.
